# A cross-sectional study on the mental health of healthcare workers treating COVID-19 positive patients in Gauteng, South Africa

**DOI:** 10.11604/pamj.2025.52.147.43262

**Published:** 2025-12-08

**Authors:** Berushka Padayachee, Diantha Pillay, Shalin Bidassey-Manilal

**Affiliations:** 1 Department of Environmental Health, Faculty of Health Science, University of Johannesburg, P.O. Box 524, Johannesburg 2006, South Africa,; 2 IPM South Africa NPC, An Affiliate of the Population Council, Johannesburg, South Africa

**Keywords:** COVID-19, healthcare worker, mental health symptom, depression, anxiety, insomnia, psychosocial support, South Africa

## Abstract

**Introduction:**

the COVID-19 pandemic had a devastating impact on society, with healthcare workers (HCWs) on the frontline bearing the brunt. As such, HCWs directly involved in the diagnosis, treatment, and care of patients with COVID-19 were at risk of developing psychological distress and other mental health symptoms. The overall aim of this study was to assess mental health symptoms among HCWs treating patients exposed to COVID-19 in Gauteng, South Africa. This research was critically important to determine the physiological burden on HCWs in response to the pandemic, so that mental health responses to the pandemic by HCWs, and psychosocial support structures in place for HCWs in South Africa can be considered.

**Methods:**

a cross-sectional study was conducted among frontline HCWs in the Inner City and Johannesburg South region (region F) of Gauteng, South Africa. Data was collected through both an online and an in-person approach. All participants completed a questionnaire enquiring about their socio-demographic characteristics and mental health symptoms. The presence and severity of symptoms of depression, anxiety, and insomnia of HCWs exposed to COVID-19 were assessed using the patient health questionnaire-9 (PHQ-9), the generalised anxiety disorder-7 (GAD-7), and the insomnia severity index-7 (ISI-7). Data analysis was performed using SPSS version 26.0 statistical software.

**Results:**

a total of 234 out of 260 contacted individuals completed the survey. The majority of the participants were female (n=190, 81.2%), aged between 36 and 45 years (n=79, 33.8%), were unmarried (n=105,44.9%), and had a tertiary education (n=217, 92.7%). The majority of the participants worked in the public sector (151, 64.5%), while the minority (n=83, 35.5%) worked in the private sector. A considerable proportion of HCWs reported symptoms of depression (n=102, 43.6%), anxiety (n=105, 44.9%), and insomnia (n=70, 29.9%). It was found that there were no significant differences in symptoms of depression (X^2^=4.8, p>0.05), anxiety (X^2^=0.8, p>0.05), and insomnia (X^2^=2.1, p>0.05) between public and private sector HCWs. The results further showed that 49.1% of the participants indicated that psychological services did exist in their place of work, and 50.9% said they did not exist. Perceptions that psychological services existed were more common among HCWs in private healthcare facilities (66.3%) than among HCWs in public healthcare facilities (39.7%). Psychosocial support systems such as mental health counselling, support groups, psychological services, and employee assistance programmes were identified as some of the key services requested by HCWs.

**Conclusion:**

the prevalence of depression, anxiety, and insomnia was shown in HCWs in Gauteng, South Africa, and there were differences in perceptions of the psychosocial support systems that exist in the region between private and public sector HCWs. The results indicate that a considerable proportion of HCWs have depression, anxiety, or insomnia, with only half indicating the presence of workplace psycho-social support structures. Psycho-social support structures for HCWs need to be strengthened and made more visible.

## Introduction

In December 2019, a pneumonia outbreak occurred in Wuhan City, Hubei Province, China, and was attributed to a novel coronavirus, severe acute respiratory syndrome coronavirus 2 (SARS-CoV-2) [1]. The WHO subsequently called the infection coronavirus disease 2019 (COVID-19), and the outbreak was declared a public health emergency of international concern (PHEIC) [1]. There was tremendous pressure on healthcare services due to the increasing spread of the COVID-19 virus, leading to high hospitalization rates and an overwhelming workload. Healthcare workers, especially those on the frontline who treat COVID-19 patients, are susceptible to developing psychological health issues [2].

Many factors, such as the increased risk of infection, considerable public attention, very high workloads, and a lack of support, may contribute to the adverse mental health symptoms experienced by HCWs [2]. Healthcare workers may have also experienced feelings of anxiety caused by the shortage of personal protective equipment (PPE) and not being able to adequately care for their children and families, due to their long working hours and heavy workload [3]. Lai *et al*. [4] established a relationship between mental health symptoms and being an HCW on the frontline of the COVID-19 pandemic and concluded that the protection of the mental health of HCWs should form part of public health interventions and is necessary to tackle the COVID-19 pandemic. The psychological impacts that pandemics have on HCWs are significant, which highlights the need for appropriate psychological support and interventions. There is a need for countries to strengthen their response to any pandemic outbreak and to consider the psychosocial support structures in place for HCWs at the frontline of the pandemic.

**Objectives:** the overall aim of this study was to assess the presence and severity of symptoms of depression, anxiety, and insomnia of HCWs exposed to COVID-19 whilst treating patients. In addition, current workplace psychosocial support systems in place to support HCWs were examined, and the relationship between these workplace psychosocial support systems and the HCWs´ sector of employment (private or public) was determined.

## Methods

**Study design and study setting:** a cross-sectional mixed-method study was conducted among HCWs in Region F (Inner City and Johannesburg District) from May 2021 to May 2022 in Gauteng, South Africa. Cross-sectional studies are usually used to define the burden of disease [5]. The study was able to measure the prevalence of depression, anxiety, and insomnia among the study sample. This site was chosen because of the high incidence of COVID-19 cases in the province and region F, specifically compared to other regions (Table 1) [6].

**Table 1:** a comprehensive analysis of COVID-19 case distribution and recovery rates by subdistrict in Johannesburg from 2020 to 2022

Breakdown by sub-district					
District	District total	District recoveries	Sub-district	Sub-district total	Sub-district recoveries
**City of Johannesburg**	154,137	147,945	**Region A:** Diepsloot, Kya Sands, Dainfern, Midrand, Lanseria, Fourways, Ivory Park	18,646	18,045
			**Region B:** Randburg, Rosebank, Emmarentia, Greenside, Melville, Mayfair, Northcliff, Parktown, Parktown North	19,763	19,034
			**Region C:** Roodepoort, Constantia Kloof, Northgate, Florida, Bram Fischerville	19,482	18,767
			**Region D:** Doornkop, Soweto, Dobsonville, Protea Glen	31],734	29,964
			**Region E:** Alexandra, Wynberg, Sandton, Orange Grove, Houghton	25,533	24,712
			**Region F:** Inner City, Johannesburg South	25,680	25,676

**Participants and data collection:** frontline HCWs (doctors and nurses specifically) in 40 private general practitioners (GPs) practices, 4 private hospitals, and 13 public healthcare facilities in the Inner City and Johannesburg South regions (region F) of Gauteng, South Africa, were approached to participate in this study. The sampling strategy that was used in this study was homogeneous purposive sampling [6], where participants were intentionally or deliberately selected based on specific shared characteristics that align closely with the research objectives. This strategy was chosen to ensure that all participants had comparable and relevant experience, namely, prolonged direct clinical exposure to COVID-19-positive patients during the pandemic in Gauteng, South Africa. This is particularly useful when you want to focus on a specific subgroup of a population to obtain rich, in-depth data about their shared experiences [6]. The target population comprised frontline healthcare workers (HCWs), including medical doctors, professional nurses, enrolled nurses, and allied health professionals (e.g., physiotherapists, radiographers, and clinical associates) who worked in designated COVID-19 wards or units across selected public sector hospitals in Gauteng.

**The inclusion criteria were as follows:** currently employed in medical practices, private hospitals, and public healthcare facilities in Gauteng that admit and treat COVID-19 positive patients; direct involvement in the clinical care or management of confirmed COVID-19 patients; a minimum of three months of continuous service in COVID-19 units during the first and/or second waves of the pandemic; aged 18 years or older; and willing and able to provide informed consent.

**The exclusion criteria were:** healthcare workers in purely administrative, managerial, or support roles without direct patient contact, workers on extended leave during the study period, and workers who refused to participate in this study. To operationalize this sampling approach, information on the participants was obtained from the management department at each chosen healthcare facility. Voluntary participation in the survey was requested from this group. To prepare for data collection, physical meetings were held with the operations manager of each clinic at a date and time convenient to them. The purpose of these meetings was to discuss what was required from the participants and to address any questions they might have regarding how to complete the survey. A suitable date was then provided by the operations managers to interview their staff on an individual basis, and each interview lasted approximately 15 minutes. Where physical meetings took place, strict COVID-19 precautions were practised by the researcher, and they were always adhered to. A face mask was worn, and social distancing, handwashing, and sanitizing were practiced at all times.

### Step-by-step sampling process

**Identification of facilities:** all medical practices, private hospitals, and public healthcare facilities were selected based on COVID-19 case burden, accessibility, and institutional approval.

**List generation:** managers provided access to staff duty rosters and deployment schedules that indicated which HCWs had been assigned to COVID-19 patients.

**Screening for eligibility:** the researcher screened lists of healthcare staff against the study´s inclusion/exclusion criteria. Where necessary, unit managers verified the nature and duration of staff deployment.

**Recruitment procedure:** eligible participants were contacted through institutional communication platforms such as internal email systems and WhatsApp workgroups already in use by the department of the facility; printed participant information sheets and consent forms were distributed by a liaison nurse or the researcher during scheduled staff meetings, morning handovers, or breaks, with appropriate social distancing in place; for those who could not attend in person, the information and consent documents were emailed or shared electronically via WhatsApp, with instructions for digital consent or safe return of hard copies.

**Data collection:** participants completed a self-administered structured questionnaire either in hard copy or electronically, depending on their preference. Data collection was conducted in private staff rooms or online using secure survey platforms. Any questions the participants had regarding how to complete the survey were addressed. Where physical meetings took place, the researcher practiced strict COVID-19 precautions and adhered to them at all times. The online approach included distributing a questionnaire to participants via an online survey hosted on REDCap. The information was loaded online and distributed to the selected healthcare facilities through a link that was provided to the participants via email. Homogeneous purposive sampling was suitable for this study because it allowed the selection of a specific group of healthcare workers who were exposed to similar occupational stressors, working under similar conditions, and sharing similar risk environments, which helped isolate the mental health outcomes related to COVID-19 clinical care exposure. The study data were collected using a structured questionnaire, which included validated scales adopted by researchers Johnson *et al*. [7], Kroenke *et al*. [8], and Morin *et al*. [9]. The questionnaire took approximately 15 minutes to complete and was structured in six sections. Section 1 solicited demographic information; section 2 enquired about medical history; sections 3, 4, and 5 included validated scales measuring depression [patient health questionnaire-9 (PHQ-9)], anxiety [generalized anxiety disorder-7 (GAD-7)], and insomnia [insomnia severity index-7 (ISI-7)]; and section 6 asked about psychosocial support systems.

**Study size:** the sample size for this study was calculated using Epi Info version 7.2. The sample size was determined using 80% statistical power, with a 95% confidence interval, assuming a two-tailed 5% alpha level of significance. After adding 25% contingency to each group for losses and multiple comparisons, the sample size was estimated to be 260 participants.

**Data management:** data were collected through self-administered questionnaires, available in both hard copy and electronic formats. Each participant was assigned a unique, anonymized code to maintain confidentiality. Completed forms were securely stored, paper forms in locked cabinets, and electronic data in password-protected, encrypted platforms. Data entry involved double-checking for accuracy and was conducted using Excel and SPSS. The dataset was cleaned to address missing values, inconsistencies, and outliers. All identifying information was removed to protect participant privacy, and access to data was restricted to the researcher, statistician, and her supervisor. In compliance with ethical guidelines and South Africa´s POPIA regulations, all data will be retained securely for five years before being permanently destroyed.

**Variables:** the dependent variables included symptoms of depression (measured by the PHQ-9 scale), anxiety (measured by the GAD-7 scale), and insomnia (measured by the ISI scale) in participants; exposure to COVID-19; and the independent variables covered sex, age, marital status, educational level, and type of healthcare facility.

**Bias:** recall bias was minimised by careful selection of the research questions, ensuring privacy so that participants felt comfortable sharing their experiences without external influences. The questionnaire was completed anonymously, allowing participants to express themselves freely to prevent biased answers, and the responses were not disclosed to other participants.

**Quantitative variables and statistical methods:** to ensure reliability, a pilot study was conducted by distributing the questionnaire to 26 participants, which constituted 10% of the sample size. This was essential, as the questionnaire needed to undergo a trial run to see if participants could understand it before it was administered. The results from the tests were consistent, the questionnaire was considered easy to complete and not time-consuming, and the questions were easy to understand.

The variables that were used in the data analysis were the following: the symptom variables of depression (measured by the PHQ-9 scale), anxiety (measured by the GAD-7 scale) and insomnia (measured by the ISI scale) in participants; exposure to COVID-19; and the confounding variables of sex, age, marital status, educational level and type of healthcare facility. Complete case (CC) analysis was performed by case-wise deletion. Any observation that had a missing value for a variable was automatically discarded, and only complete observations were analysed. Descriptive and inferential analyses were conducted. Frequencies, percentages, and standard deviations were calculated.

The presence and severity of symptoms of depression, anxiety, and insomnia of HCWs exposed to COVID-19 were assessed using the PHQ-9, GAD-7, and ISI-7. The total scores of these measurement tools were interpreted as follows: PHQ-9: normal depression (0-4), mild depression (5-9), moderate depression (10-14), and severe depression (15-21); GAD-7: normal anxiety (0-4), mild anxiety (5-9), moderate anxiety (10-14), and severe anxiety (15-21); ISI: normal insomnia (0-7), sub-threshold insomnia (8-14), moderate insomnia (15-21), and severe insomnia (22-28) [4]. The cut-off scores for detecting symptoms of major depression, anxiety, and insomnia were 10, 7, and 15, respectively. Participants who had scores greater than the cut-off threshold were characterised as having severe symptoms.

A Chi-squared test was used to compare the odds of severe depression, anxiety, and insomnia of healthcare workers between private and public sector healthcare facilities. The purpose of the Chi-squared test is to determine if a difference between observed data and expected data is due to chance or if it is due to a relationship between the variables that are being studied, in this case, mental health symptoms of HCWs between the public sector and the private sector [10]. The psychosocial support systems in place to support HCWs at the frontline of the COVID-19 pandemic were examined using a semantic thematic analysis. Themes were defined by the services that HCWs reported on in response to the question regarding what type of workplace psychosocial services exist in their workplace. The question that gathered the qualitative data that was used for the thematic analysis was a 2-part question: “In your place of work, do you have social or psychological services that exist? If yes, please specify what these services are”. The relationship between psychosocial support systems and the HCW´s sector of employment (private or public) was also determined using a Chi-squared test. “Yes” was coded as (1) and “No” was coded as (2). Data analysis was performed using SPSS version 26.0 statistical software. The significance level was set at α = 0.05, and all tests were two-tailed. P-value of < 0.05 were considered statistically significant.

**Ethics approval and consent to participate:** ethical approval to conduct this study was granted by the University of Johannesburg´s Higher Degrees Committee and its Research Ethics Committee (REC-973-2021). Permission to participate in the study was requested from the relevant authorities, and approval from these facilities was granted. Participants were given an information letter with details of the study as well as a consent letter before the commencement of the study. Those not willing to participate were given the right to do so. Confidentiality of responses was also ensured throughout the research process. All personal information about the research participants was collected, stored, used, and destroyed in ways that respected the privacy and confidentiality of the participants. Electronic data was stored on the REDCap system, on a laptop that was only accessible to the researcher through access control, and which required a username and password to log on to the system. Since the research was conducted solely, only the researcher had access to the data on the laptop.

## Results

**Participants and descriptive data:** of the 260 healthcare workers approached to participate in the study, 234 respondents (90%) completed the survey. The majority of the respondents in the study (33.8%, n=79) were aged between 36 and 45 years, 31.6% (n=74) were aged between 25 and 35 years, 18.8% (n=44) were between the ages of 46 and 55, 15.4% (n=36) were 56 years or older, and 0.4% (n=1) were aged between 18 and 24 years. The majority of the participants were female (81.2%, n=190), while 18.8% (n=44) of the sample were male. Regarding the participants´ marital status, 44.9% (n=105) of the sample were unmarried, 38.9% (n=91) were married, 8.1% (n=19) were divorced, 3.4% (n=8) were widowed, and 3% (n=7) were separated. The vast majority of the HCWs had a tertiary education (92.7%, n=217). Of the 234 responding participants, 151 (64.5%) were in the public sector, and 83 (35.5%) were in the private sector (Table 2).

**Table 2:** demographic characteristics of the study participants who participated in a study on the Mental Health of Healthcare Workers treating COVID-19 positive patients in Johannesburg, South Africa

	Participants from the public sector (N=151)		Participants from the private sector (N=83)		Total (N=234)	
	N	%	N	%	N	%
**Age (years)**						
18-24	0	0%	1	1.2%	1	0.4%
25-35	58	38.4%	16	19.3%	74	31.6%
36-45	52	34.4%	27	32.5%	79	33.8%
46-55	24	15.9%	20	24.1%	44	18.8%
56+	17	11.3%	19	22.9%	36	15.4%
**Missing data**						
Gender						
Male	30	19.9%	14	16.9%	44	18.8%
Female	121	80.1%	69	83.1%	190	81.2%
**Marital status**						
Unmarried	77	50.9%	28	33.7%	105	44.9%
Married	57	37.7%	34	40.9%	91	38.9%
Separated	3	1.9%	4	4.8%	7	3.0%
Divorced	10	6.6%	9	10.8%	19	8.1%
Widowed	4	2.6%	4	4.8%	8	3.4%
**Missing data**						
Educational level						
Secondary	12	7.9%	5	6%	17	7.3%
Tertiary	139	92.1%	78	94%	217	92.7%

**Presence and severity of depression, anxiety, and insomnia in participants:** the results for the PHQ-9 scale measuring depression indicated that 43.6% (n=102) of the HCWs who participated in the study showed symptoms of depression. When categorized by severity of symptoms, 24.4% (n=57) of the HCWs showed symptoms of mild depression, 14.1% (n=33) had symptoms of moderate depression, and 5.1% (n=12) had symptoms of severe depression (Table 3). The results for the GAD-7 scale measuring anxiety indicated that 44.9% (n=105) of the HCWs showed symptoms of anxiety. When categorized by severity of symptoms, 26.9% (n=63) of the HCWs showed symptoms of mild anxiety, 10.7% (n=25) had symptoms of moderate anxiety, and 7.3% (n=17) had symptoms of severe anxiety (Table 3). The results for the ISI scale measuring insomnia showed that 30.4% (n=71) of HCWs displayed symptoms of insomnia. When categorized by severity of symptoms, 19.7% (n=46) of HCWs showed symptoms of sub-threshold insomnia, 8.1% (n=19) had symptoms of moderate insomnia, and 2.6% (n=6) had symptoms of severe insomnia (Table 3).

**Table 3:** the results for the patient health questionnaire - 9 scale measuring mental health symptoms of depression, anxiety and insomnia in Johannesburg, South Africa

The PHQ-9 scale measuring depression	Frequency (n)	Percentage (%)
Normal (0-4)	132	56.4
Mild depression (5-9)	57	24.4
Moderate depression (10-14)	33	14.1
Severe depression (15-21)	12	5.1
Total	234	100.0
**GAD-7 scales measuring anxiety**		
Normal anxiety (0-4)	129	55.1
Mild anxiety (5-9)	63	26.9
Moderate anxiety (10-14)	25	10.7
Severe anxiety (15-21)	17	7.3
**Total**	234	100.0
**ISI-7 scale measuring insomnia**		
Normal insomnia (0-7)	163	69.7
Sub-threshold insomnia (8-14)	46	19.7
Moderate insomnia (15-21)	19	8.1
Severe insomnia (22-28)	6	2.6
Total	234	100.0

* PHQ (patient health questionnaire) *GAD (generalised anxiety disorder) *ISI (insomnia severity index)

**Relationship between mental health symptoms of HCWs and their sector of employment:** there were no significant differences in symptoms of depression (X^2^=4.28, p>0.05), anxiety (X^2^=0.79, p>0.05), and insomnia (X^2^=2.17, p>0.05) between public and private healthcare facilities as shown by the chi-square tests (Table 4, Table 5, Table 6).

**Table 4:** association of the patient health questionnaire - 9 total score and type of healthcare facility in Johannesburg, South Africa

Symptoms of depression		Type of healthcare facility			Pearson chi-square	Asymptotic significance (two-sided)
PHQ-9 scale		Public	Private	Total		
Normal depression (0-4)	Count (n)	82	50	132	4.824*	0.185
	% within the type of healthcare facility	54.3%	60.2%	56.4%		
Mild depression (5-9)	Count (n)	41	16	57		
	% within the type of healthcare facility	27.2%	19.3%	24.4%		
Moderate depression (10-14)	Count	23	10	33		
	Count (n)	23	10	33		
	% within the type of healthcare facility	15.2%	12.1%	14.1%		
Severe depression (15-21)	Count (n)	5	7	12		
	% within the type of healthcare facility	3.3%	8.4%	5.1%		
Total	Count (n)	151	83	234		
	% within the type of healthcare facility	100.0%	100.0%	100.0%		

* Significant at 5% level *PHQ (patient health questionnaire)

**Table 5:** contingency table of generalised anxiety disorder-7 total score and type of healthcare facility in Johannesburg, South Africa

Symptoms of anxiety		Type of healthcare facility			Pearson chi-square	Asymptotic significance (two-sided)
GAD-7 scales		Public	Private	Total		
Normal anxiety (0-4)	Count (n)	85	44	129	0.791*	0.852
	% within the type of healthcare facility	56.3%	53.0%	55.1%		
Mild anxiety (5-9)	Count (n)	39	24	63		
	% within the type of healthcare facility	25.8%	28.9%	26.9%		
Moderate anxiety (10-14)	Count (n)	15	10	25		
	% within the type of healthcare facility	9.9%	12.1%	10.7%		
Severe anxiety (15-21)	Count (n)	12	5	17		
	% within the type of healthcare facility	7.9%	6.0%	7.3%		
Total	Count (n)	151	83	234		
	% within the type of healthcare facility	100.0%	100.0%	100.0%		

* Significant at 5% level *GAD (generalised anxiety disorder)

**Table 6:** association between insomnia severity index total score and type of healthcare facility where the study was conducted to determine the mental health of workers during COVID-19

Symptoms of insomnia		Type of healthcare facility			Pearson chi-square	Asymptotic significance (two-sided)
ISI scale		Public	Private	Total		
Normal insomnia (0-7)	Count (n)	106	57	163	2.171*	0.704
	% within the type of healthcare facility	70.2%	68.7%	69.7%		
Sub-threshold insomnia (8-14)	Count (n)	27	19	46		
	% within the type of healthcare facility	17.9%	22.9%	19.7%		
Moderate insomnia (15-21)	Count (n)	14	5	19		
	% within the type of healthcare facility	9.3%	6.0%	8.1%		
Severe insomnia (22-28)	Count (n)	4	2	6		
	% within the type of healthcare facility	2.6%	2.4%	2.6%		
Total	Count (n)	151	83	234		
	% within the type of healthcare facility	100.0%	100.0%	100.0%		

* Significant at 5% level *ISI (insomnia severity index)

**Examination of psychosocial support systems in place to support HCWs at the frontline of the COVID-19 pandemic:** to assess the existence of social or psychological services in the HCWs´ place of employment, HCWs in both the public and private sectors were asked if social or psychological services existed in their place of work. In total, 49.1% (n=115) of the HCWs indicated that these services are provided in their place of work, while 50.9% (n=119) indicated that they are not. The results demonstrate that there are statistically significant differences (X^2^=15.08, p<0.05) in the responses between private and public sector HCWs regarding their perceptions of whether social and psychological services existed at the time of the study (Table 7). This suggests that perceptions that social and psychological services existed were associated with the type of healthcare facility. Perceptions that social and psychological services existed were more common among HCWs in private healthcare facilities (66.3%, n=55) than among HCWs in public healthcare facilities (39.7%, n=60). Perceptions that social and psychological services did not exist were more common among HCWs in public healthcare facilities (60.3%, n=91) than among HCWs in private healthcare facilities (33.7%, n=28).

**Table 7:** an evaluation on the availability of social or psychological services in relation to the type of healthcare facility accessible to both public and private healthcare workers during the COVID-19 pandemic in Gauteng, South Africa

		Type of healthcare facility			Pearson chi-square	Asymptotic significance (two-sided)
	In your place of work, do you have social or psychological services that exist?	Public	Private	Total		
Yes	Count (n)	60	55	115	15.08*	0.000*
	% within the type of healthcare facility	39.7%	66.3%	49.1%		
No	Count (n)	91	28	119		
	% within the type of healthcare facility	60.3%	33.7%	50.9%		
Total	Count (n)	151	83	234		
	% within the type of healthcare facility	100.0%	100.0%	100.0%		

* Significant at 5% level

**Figure 1:**
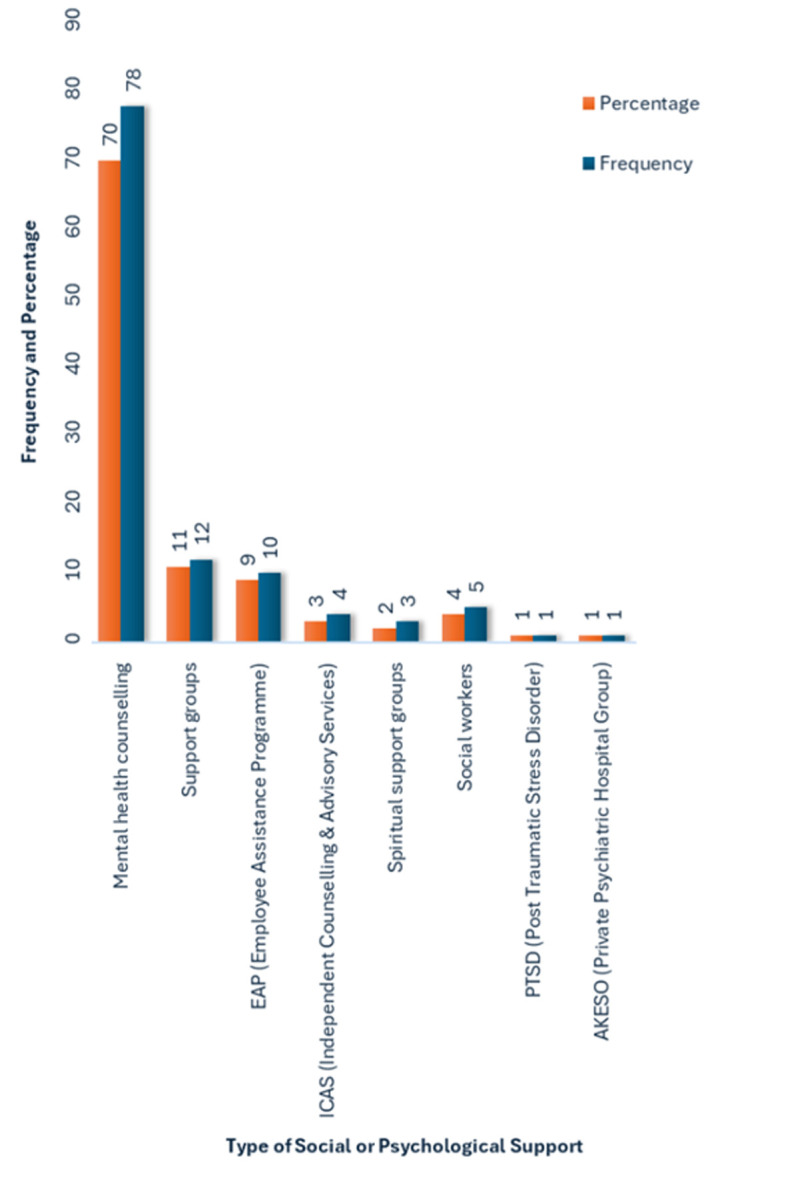
types of social or psychological services available in public and private hospitals, in Gauteng, as indicated by healthcare workers (2022, N=115)

**Types of social or psychological services perceived by the HCWs in their place of employment:** the HCWs were asked an open-ended question regarding the specific types of social or psychological services that were provided in their place of work. Of those who reported having these services (49.1%, n=115), mental health counselling (MHC) dominated the responses and was cited by 69.5% (n=80) of the HCWs (Figure 1). However, 9 of the respondents noted that mental health counselling services are insufficient or not visible, and this emerged as the main theme based on the responses of the HCWs. The public healthcare sector respondents #245 and #246, aged 40 and 45 years, respectively, commented that MHC is not seen by them. The same sentiment was expressed by a private healthcare sector respondent #239, aged 32, who stated that “*MHC is not visible*”. Public sector respondent #247, aged 36, stated that “*MHC services are not visible and are only available on paper*” and asserted that “*visible, active and acceptable services are needed*”. This highlights the gap in mental health service provision to HCWs.

Support groups were another service cited by the HCWs (11.3%, n=13). A private sector respondent #220, aged 39, reiterated the theme above, namely that support groups, like mental health counselling services, are not adequate, and additional services were requested. Employee assistance programme (EAP) services also emerged as a psychological service provided, cited by 7.8% (n=9) of the HCWs. However, respondent #105, aged 33 years, indicated that the EAP services that were supposed to be provided were not available in their public health facility. This respondent commented, “*EAP is never seen or interacted with. Need mental health care services to be provided*.”

Only 3.5% (n=4) of the HCWs indicated that independent counselling and advisory services (ICAS) were available to them. A private sector respondent #235, aged 46 years, mentioned that ICAS was not visible. Only 2.6% of the HCWs (n=3) indicated that spiritual support groups were available to them in their place of work. Social workers were cited by 3.5% (n=4) of HCWs in the survey as a psychological service available in their place of work. However, a public sector respondent #80, aged 31 years, indicated that although social workers were provided, they were not available: “*We don´t see the social workers. Need mental health counselling to be provided*.” PTSD support services and Akeso, which is a psychiatric rehabilitation hospital for acute mental illness and substance abuse, were services that emerged as psychological services provided to the HCWs. However, each was cited by only 0.9% of HCWs in the survey, and they did not feature prominently.

As mentioned, more than half of the HCWs in the study (50.9%, n=119) indicated that psychological services did not exist in their place of work. When asked if they would like these services to be provided to them, 30.8% (n=72) indicated that they wanted these services to be provided. Counselling and debriefing emerged as the service requested the most by the HCWs, mentioned 37 times. Support groups were the second-most requested service, mentioned 14 times, followed by psychological services (mentioned 10 times), spiritual support groups (mentioned five times), PTSD support services (mentioned five times), EAP services (mentioned five times), and social workers (mentioned four times).

## Discussion

**Presence and severity of depression, anxiety, and insomnia in participants:** this study provides insight into the mental health symptoms displayed by frontline HCWs from both public and private healthcare facilities in South Africa. The damaging impact of previous pandemics on the mental health of HCWs has been well documented. Al Ghobain *et al*. [11]; Chen *et al*. [12]; Khalid *et al*. [13], and Sin *et al*. [14] reported high levels of depression, anxiety, and post-traumatic stress disorders among HCWs during the 2003 SARS pandemic and the 2015 MERS outbreak. Chen *et al*. [15] reported that frontline HCWs, general HCWs, and the general population in 12 African countries showed an overall prevalence of mental health disorders of 49%, 36% and 38%, respectively. These findings are similar to those of this study, which showed that 43.6%, 44.9% and 29.9% of HCWs reported symptoms of depression, anxiety, and insomnia, respectively. There were no significant differences between these mental health symptoms and the sector of employment (public or private) among the HCWs. This could be attributed to the differences in the sample size of participants, as more responses were collected from HCWs in the public sector (151) than in the private sector (83). Almost one-fifth (19.2%) of participants suffered from moderate to severe depression, 17.9% of participants suffered from moderate to severe anxiety, and 10.7% of participants reported moderate to severe insomnia. These findings are consistent with those of Dawood *et al*. [16] who reported high levels of depression (51%), anxiety (47.2%), and post-traumatic stress (44.3%) in public sector doctors and nurses employed in KwaZulu-Natal during the COVID-19 pandemic, with 16.2%, 21.3% and 16.2% in the moderate to severe range, respectively.

Other studies conducted by Afulani *et al*. [17], Ali *et al*. [18], Keubo *et al*. [19], Kwobah *et al*. [20] and Olashore *et al*. [21], which examined the impact of COVID-19 on HCWs in Africa, have shown a prevalence of anxiety of mild, moderate, and severe levels ranging from 25.5% to 90.5%, and a prevalence of depressive symptoms ranging from 32.1% to 94.0%. Most of these studies used the GAD-7 and the PHQ-9 to determine anxiety and depression, respectively. Hoque *et al*. [22] demonstrated a high prevalence of stress (97.5%), anxiety (97.5%), and depression (44%) disorders among nurses at a primary health care (PHC) facility in Durban, South Africa. The findings of this study are also comparable with those of Shah *et al*. [23], who reported symptoms of depression (53.6%), anxiety (44.3%), and insomnia (41.1%) among HCWs in COVID-19 wards in one government and two private hospitals in Kenya. In China, Lai *et al*. [4] demonstrated considerable levels of depression (50.4%), anxiety (44.6%), and insomnia (34.0%) among HCWs who treated COVID-19-positive patients, which are similar to the findings of this study.

There are several reasons why mental health symptoms such as depression, anxiety, and insomnia may arise in HCWs during a pandemic. With a pandemic comes feelings of uncertainty and fear of the disease, loss of loved ones, financial difficulties, and isolation [24]. Healthcare workers have to contend with very high workloads, limited resources and equipment, and physical discomfort due to wearing PPE. In addition, when the initial outbreak of COVID-19 occurred in South Africa, very little was known about the virus, and no established protocols or evidence-based clinical treatments existed at the time. In August 2020, there were 589,886 cases of COVID-19 and 11,982 deaths from COVID-19 in the general population of South Africa, and the number of infected HCWs was 27,360, of which 78% were from the public sector, and 22% from the private sector, with 240 deaths reported [22]. There would have been anxiety and concerns among HCWs about contracting the virus and transmitting it to their loved ones, and also the fear of isolation from family and friends, and a lack of control over the situation. All these factors are likely to compound the incidence of psychological problems experienced by HCWs in South Africa, such as fear, anxiety, depression, stress, and insomnia [23]. It is also important to consider that these factors are separate from the conditions specific to the nature of the work of health professionals on the African continent, namely, high levels of stress, job dissatisfaction as a result of poor remuneration, increased job demands, and long working hours [22]. These factors may contribute to the prevalence of anxiety, depression, and insomnia in this population of frontline HCWs [24].

**Assessment of psychosocial support systems for HCWs in their place of employment:** Thobane *et al*. [25] showed that frontline nurses in the Tshwane district of Gauteng requested psychosocial support and counselling during the COVID-19 pandemic. These nurses requested that the hospital management, together with the Department of Health (DoH), provide psychologists and social workers for them and their families. Evidence of the effectiveness of psychosocial support systems was shown by Zingela *et al*. [26], who developed a psychological preparedness training programme to support frontline HCWs in three resource-limited hospitals in South Africa dealing with the COVID-19 outbreak. They supported 761 healthcare workers during the 20 weeks of the programme. Their results demonstrated a significant positive change from before the intervention to after it in the following: the perceptions of HCWs regarding the outbreak, their anxiety associated with the outbreak, their ability to control their reaction to stress, and their perceptions of their ability to support others. The training programme proved to be beneficial to HCWs, as it improved their knowledge about the pandemic, and it gave them the confidence to handle the pressure and stress they experienced due to the increase in infections. It also gave HCWs the tools to recognise the signs of stress in others and provide support [26].

In examining the current psychosocial support systems in place for HCWs in this study, the results of this study showed that 49.1% of the participants indicated that there were psychological services that existed in their place of work, and 50.9% said there were not. The results indicated that perceptions that psychological services existed were more common among HCWs in private healthcare facilities (66.3%) than among HCWs in public healthcare facilities (39.7%). When asked what types of social or psychological services existed in their place of work, mental health counselling dominated the responses, followed by support groups, EAP services, ICAS, spiritual support groups, social workers, PTSD support, and Akeso. However, many of the HCWs commented that the psychological services that were provided were either not visible or inadequate, and they requested that more services be provided.

One respondent articulated that psychological services were not visible and were only available on paper. Another respondent mentioned that they do not see social workers, and they asked for mental health counselling. Others expressed the need for active and acceptable mental health services to be provided to them. The main theme that emerged was that the psychosocial support services that were supposed to have been provided, such as MHC, EAP, ICAS, and PTSD support, were lacking and were not visible enough, and that HCWs had insufficient interaction with these services. One HCW´s experiences of inadequate and insufficient services impacted them on a personal level as well, and they spoke of being admitted to a mental hospital for depression and being divorced soon thereafter. Many of the HCWs, all from the public sector, shared the same sentiment, namely that there is a lack of adequate services, and they expressed the need for regular debriefing sessions. Others indicated their need for PTSD support services, as they have been traumatized by the COVID-19 pandemic. A common theme verbalized by the HCWs was that more services needed to be provided, and they specified the types of services they required. Spiritual support groups, counselling and debriefing, PTSD support, and even financial counselling emerged as psychosocial services that were requested by HCWs in this study.

The results of this study highlight the disparities in the provision of and access to psychosocial support services between the public and private healthcare sectors in South Africa. These differences can be attributed to vast inequities that persist, a shortage of trained mental healthcare providers, perceptions and beliefs regarding the evidence base for mental health screening and treatment, organizational readiness and willingness to change, insufficient infrastructure and resources, and long waiting times in public healthcare facilities [24]. Key strategies to support psychological interventions in routine practice are needed to overcome implementation barriers in these facilities.

**Psychosocial interventions for HCWs in South Africa:** the COVID-19 pandemic had a profound impact on the mental health of frontline HCWs, who had to contend with physical exhaustion, incredibly high workloads, stress, fear, and paranoia. Healthcare workers were at risk of developing psychological symptoms, and they desperately needed psychosocial interventions to help them cope. South African HCWs experienced many of the challenges of shortages of staff and essential resources, grief, fear, anxiety, and distress. It is crucial to protect the mental health of our HCWs, as they are the section of the population that is needed most during a pandemic. Appropriate interventions and strategies to improve the mental health of HCWs need to be adopted in light of COVID-19 and future pandemics.

There is not enough data on psychosocial interventions for HCWs in South Africa. A systematic review concluded that there is inadequate evidence from studies carried out during or after disease pandemics that can advise on the choice of interventions and measures that are beneficial to the mental health of HCWs [27]. This study showed that there was a vast difference between the private and the public sectors in perceptions of whether psychological services are provided for HCWs. Services such as MHC, EAP, and ICAS, which are designed for frontline workers in the healthcare sector, were shown to be invisible, inadequate, and insufficient. There is a clear gap in the provision of mental health services for HCWs, especially in the public sector.

Psychosocial support, including other interventions, must target high-risk groups, such as HCWs and other first responders [28]. Evidence that these interventions work was demonstrated during the Ebola outbreak, as HCWs who were provided with strong support systems and psychological interventions showed reduced adverse mental health symptoms [29]. It is important to institute measures to foster resilience, proactively monitor mental wellness, and address work and health concerns, as these measures will be fundamental to delivering high-quality, safe, and effective care. Providing HCWs with effective and consistent psychosocial support is necessary both during and after the COVID-19 pandemic.

Rana *et al*. [30] recommend that a detailed psychological crisis intervention plan be developed “a) by building a mental health intervention medical team to provide online courses for awareness of the psychological impact of stressful events to guide medical workers, b) and a psychological assistance hotline intervention for medical workers to discuss their psychological concerns with the trained and specialized team of mental health practitioners”. They suggest that hospitals should implement a frequent shift system, guarantee food and living supplies, and offer pre-job training to address the identification of and responses to psychological issues in patients, families, and themselves. They also stress that psychological counsellors/counselling psychologists should regularly visit HCWs to provide support. They recommend that an online psychological crisis intervention model (PCIM) be developed and implemented to handle the secondary mental health problems encountered during the COVID-19 pandemic. They suggest that the PCIM should consist of teams of physicians, psychiatrists, psychologists/mental health practitioners, and social workers, to deliver early psychological intervention to patients, families, and medical staff.

In Gauteng, the Department of Health´s research and evaluation team requested evidence-based recommendations regarding the impact of the COVID-19 pandemic on the mental health of HCWs. Robertson *et al*. [31] identified interventions and strategies in this regard and made a presentation to the senior leadership of the Gauteng Department of Health´s COVID-19 response team in April 2020. They suggested that (1) the institution and management are fundamental to the response and that (2) accessible and appropriate psychological support is needed. Their suggestions were aligned with WHO guidelines for HCWs in public health emergencies and for ensuring mental health in the workplace. They stipulated that the responsibilities of the institution should include delivery of adequate health infrastructure, infection prevention and control, workforce staffing appropriate for the caseload, and flexible working hours. However, many challenges need to be addressed for this to be implemented in sub-Saharan Africa.

**Interpretation and generalisability:** strong leadership and communication strategies are vital, and psychosocial care and proper training of management staff must be a priority. An individualized approach should be taken for HCWs with severe symptoms. Private and volunteer associations should also become involved to supplement the mental health professionals, who are in short supply. Mental health and psychosocial support are relevant to many other domains of government, including health, protection and social services, nutrition, labour, education, and justice [32]. Mental health support systems should be fully considered across the government´s health, social, and economic responses and recovery plans during the COVID-19 pandemic. These systems should reach the most vulnerable and those most affected, especially the frontline HCWs, who, in ensuring the care and well-being of their patients, risk their lives every day by being exposed to this virus. Mental health will always remain a concern, even beyond any pandemic. As our own national Mental Health Policy Framework highlights, “it costs South Africa more to not treat mental illness than to treat it” [33].

**Limitations:** this study has several limitations. Selective memory bias of participants could have occurred since they were asked to remember experiences or events that had happened in the past. Social desirability bias was also possible, as some HCWs may not have answered truthfully regarding their mental health status. This study was cross-sectional in nature, and, as such, it is unable to account for potential changes in psychological disorders over a long period. The number of HCWs who participated in this survey was limited, which limits the generalizability of the results. Finally, a major limitation is that more responses were collected from HCWs in the public sector than in the private sector, due to many challenges and restrictions that were experienced during data collection. In the private sector, some challenges were experienced where access was limited or, at times, restricted to many facilities, hence there was a lack of sufficient responses. This resulted in an unequal distribution of the sample.

## Conclusion

Mental health services for HCWs should be a standard part of care, given the considerable pressure and stress of their jobs. Wide-ranging psychosocial intervention models targeted at HCWs should be developed and executed effectively, and they should include individual and organisational approaches. These approaches, if executed effectively, can alleviate the psychological impact that COVID-19 has had on the population of HCWs in South Africa, and they can improve mental health symptoms. The integration of mental health services into primary healthcare is critical and should be prioritised. Studies such as this, which involve assessments of mental health, can support policy efforts to improve resources for mental health and inform how these resources can be targeted most efficiently. Tracking and understanding the mental health burden of the COVID-19 crisis is a public health research priority. There is a need for future longitudinal research that allows for a direct comparison of person-by-person mental health both before and throughout the pandemic using validated mental health measures. Recommendations from this study on psychosocial support structures for frontline health workers have been made, and this study presents a case for an improvement of mental health services provided to HCWs, given the perceived inadequacies that were shown.

### What is known about this topic

- The coronavirus was a global threat and an international public health emergency, and has had detrimental effects on the mental health of healthcare workers;
- Previous studies have reported the psychological effects of the 2003 SARS and 2012 MERS outbreaks on HCWs;
- The statistics on mental health conditions were already concerning before COVID-19 emerged.

### What this study adds

- A considerable proportion of healthcare workers in Gauteng experienced symptoms of depression, anxiety, and insomnia during the COVID-19 pandemic;
- Mental health distress was widespread across both public and private sectors, with no statistically significant differences in prevalence between the two groups;
- Private sector workers reported greater access to workplace psychological services, while limited psychosocial support in public hospitals coincided with high levels of mental health issues among staff.

## Available online at

https://www.panafrican-med-journal.com//content/article/52/147/full
